# Arctic marine phytobenthos of northern Baffin Island

**DOI:** 10.1111/jpy.12417

**Published:** 2016-07-07

**Authors:** Frithjof C. Küpper, Akira F. Peters, Dawn M. Shewring, Martin D. J. Sayer, Alexandra Mystikou, Hugh Brown, Elaine Azzopardi, Olivier Dargent, Martina Strittmatter, Debra Brennan, Aldo O. Asensi, Pieter van West, Robert T. Wilce

**Affiliations:** ^1^ Scottish Association for Marine ScienceDunbeg, Oban, ArgyllPA37 1QAUK; ^2^ OceanlabUniversity of AberdeenMain StreetNewburghAB41 6AAUK; ^3^ BEZHIN ROSKO40 rue des pêcheurs29250SantecFrance; ^4^ UK National Facility for Scientific DivingScottish Association for Marine ScienceDunbeg, Oban, ArgyllPA37 1QAUK; ^5^ Centre International de Valbonne190 rue Frédéric Mistral06560ValbonneFrance; ^6^ 15, rue LamblardieF‐75012ParisFrance; ^7^ Institute of Medical SciencesCollege of Life Sciences and MedicineAberdeen Oomycete LaboratoryUniversity of AberdeenForesterhillAberdeenAB25 2ZDUK; ^8^ Department of BiologyUniversity of MassachusettsAmherstMassachusetts01003USA

**Keywords:** COI, cox3, *Desmarestia*, germling emergence, macroalgae, molecular barcoding, Phaeophyceae, *Pylaiella*

## Abstract

Global climate change is expected to alter the polar bioregions faster than any other marine environment. This study assesses the biodiversity of seaweeds and associated eukaryotic pathogens of an established study site in northern Baffin Island (72° N), providing a baseline inventory for future work assessing impacts of the currently ongoing changes in the Arctic marine environment. A total of 33 Phaeophyceae, 24 Rhodophyceae, 2 Chlorophyceae, 12 Ulvophyceae, 1 Trebouxiophyceae, and 1 Dinophyceae are reported, based on collections of an expedition to the area in 2009, complemented by unpublished records of Robert T. Wilce and the first‐ever photographic documentation of the phytobenthos of the American Arctic. Molecular barcoding of isolates raised from incubated substratum samples revealed the presence of 20 species of brown seaweeds, including gametophytes of kelp and of a previously unsequenced *Desmarestia* closely related to *D. viridis*, two species of *Pylaiella*, the kelp endophyte *Laminariocolax aecidioides* and 11 previously unsequenced species of the Ectocarpales, highlighting the necessity to include molecular techniques for fully unraveling cryptic algal diversity. This study also includes the first records of *Eurychasma dicksonii*, a eukaryotic pathogen affecting seaweeds, from the American Arctic. Overall, this study provides both the most accurate inventory of seaweed diversity of the northern Baffin Island region to date and can be used as an important basis to understand diversity changes with climate change.

**Abbreviations**
COI: cytochrome c oxidase subunit I
ML: maximum likelihood
NJ: neighbor‐joining

In the Arctic and Antarctic bioregions, average temperatures are expected to rise by as much as 5°C until the end of the 21st century, twice more than the global mean (IPCC 2014). Recent decades have seen massive loss of Arctic sea ice, combined with rising sea surface temperature in the ice‐free areas in the summer – accelerating over the last decade to a loss exceeding 2 million km^2^ with a new minimum reached in 2012 (Parkinson and Comiso 2013, Kwok 2015) which is unprecedented for the last 1,450 years at least (Kinnard et al. 2011). The disappearance of Arctic summer sea ice is considered a critical tipping element for the global environment (Lenton et al. 2008) and contrasts the development in the Antarctic (Turner and Overland 2009). The transition from a high‐albedo sea ice surface to an open sea, absorbing most of the solar irradiance, is also a major factor amplifying warming in the Arctic (Serreze and Barry 2011). A recent study (Halfar et al. 2013) provided a high‐resolution, multi‐century time series documenting the decline of Arctic sea ice, using the buildup of coralline red algae as proxy. This development may have far‐reaching environmental, political, and socio‐economic impacts for the Arctic and the entire world (Hassol 2004), including the opening of new, trans‐Arctic shipping routes (Smith and Stephenson 2013) and major ecological consequences both in the sea and on land (Post et al. 2013). Sea ice (with variable snow cover) creates a spatially and seasonally very heterogeneous light environment for the light‐limited Arctic shallow benthic ecosystems at the underside of the ice (Glud et al. 2007).

Seaweeds are major primary producers and constitute significant standing stock in Arctic inshore waters. In Young Sound, a study site in NE Greenland, comparable to those in northern Baffin Island discussed here, macroalgae accounted for 23% of the fjord's primary production, compared to 16% for benthic diatoms and 60% for phytoplankton (Glud et al. 2002). At the same site, foliose macroalgae occurred in the depth range of 2–25 m. They contributed markedly to primary production in shallow water but became insignificant at water depths >15 m, while benthic diatoms contributed most to primary production at intermediate water depths (Krause‐Jensen et al. 2007). Here, at water depths greater than 30 m, only coralline algae occurred (Roberts et al. 2002, Krause‐Jensen et al. 2007).

The exploration of Arctic seaweeds was pioneered by Kjellman (1883). His “The Algae of the Arctic Sea” was the first – and until now only – work taking a holistic view of the Arctic phytobenthos. The history of Arctic seaweed exploration and the issue of describing the genuinely “Arctic” features of the seaweed biodiversity has been reviewed by Wilce (2015). Lee (1980) published the first checklist for the region; however, this includes many records from sub‐Arctic (boreal) rather than genuinely Arctic locations, shifting the focus from a strictly Arctic flora towards a wider boreal, North American flora.

Nevertheless, knowledge of the American Arctic's seaweeds is, at best, sketchy – in particular, in terms of their biodiversity, ecology, biomass and contributions to biogeochemical cycles of the Arctic. There is a pressing need for an inventory of seaweeds of the American High Arctic considering that it would constitute an important baseline dataset, which needs to be completed prior to the major environmental changes that have started in recent years. A recent study (Saunders and McDevit 2013) has highlighted the need of complementing morphology‐based identifications of macroalgae by DNA barcoding and that much of the current data, at least for the Canadian Arctic, should be used with caution – also because purely morphological approaches may miss part of the actual diversity. DNA barcoding is widely considered a suitable approach for identifying marine taxa due to lack of reliable morphological features for diagnosis (Radulovici et al. 2010).

The scope of this study was to conduct a state‐of‐the‐art identification of the macroalgal flora of the northern Baffin Island region for establishing a species checklist, based upon an expedition in summer 2009 (led by FCK) and unpublished materials from previous expeditions (led by RTW). A major driver was the consideration that there is an urgent need for such a survey for establishing an important knowledge base to understand diversity changes with the rapid climate change in the region. Our 2009 expedition aimed to complement collections of several decades, enhanced by the availability of DNA barcoding (Saunders and McDevit 2013) and algal culturing based on the Germling Emergence Method (Peters et al. 2015). The chosen study site at Cape Hatt and Ragged Channel had been the site of a major investigation about the effects of an oil spill in the Arctic marine environment, the Baffin Island Oil Spill (BIOS) project (Snow et al. 1987) which included a preliminary survey of the site's dominant seaweed species (Cross et al. 1987). Given that our data were generated in the Eclipse Sound/Ragged Channel area of northern Baffin Island, it is at present not clear to what extent they will be relevant to the wider Canadian Archipelago also considering that studies on macrofauna in the region (Cusson et al. 2007, Piepenburg et al. 2011, Goldsmit et al. 2014) have shown considerable diversity even at small spatial scales.

Also, the approach applied in this study is unprecedented for the Arctic as a whole in that it combines an array of complementary techniques. The isolation of algal cultures from sediment and other benthic substratum samples is particularly suitable for studying the algal flora of remote locations (Müller and Ramírez 1994), complementing the collection of herbarium specimens on‐site, documentation by underwater photography and filming and the evaluation of historic herbarium records, in order to capture the entire macroalgal flora. This includes its more cryptic, filamentous representatives, which are apparent only in laboratory culture. Finally, and except for a single record of *Eurychasma dicksonii* in Svalbard in the European Arctic (Jenneborg 1977), no previous study has targeted eukaryotic pathogens affecting marine algae in the Arctic region.

## Materials and Methods

A backlog of unpublished information (R.T. Wilce) of several decades contributed to formulating the research questions of this project and the preparation of the 2009 expedition. This included previous expeditions to the Cape Hatt/Ragged Channel area in August of three consecutive years, 1981, 1982, and 1983, and September 2004. The algal herbarium at UMass, established with collections from the American Arctic (Alaska to Labrador), and the study sites around Cape Hatt in particular, provided baseline material for this study.

**Figure 1:**
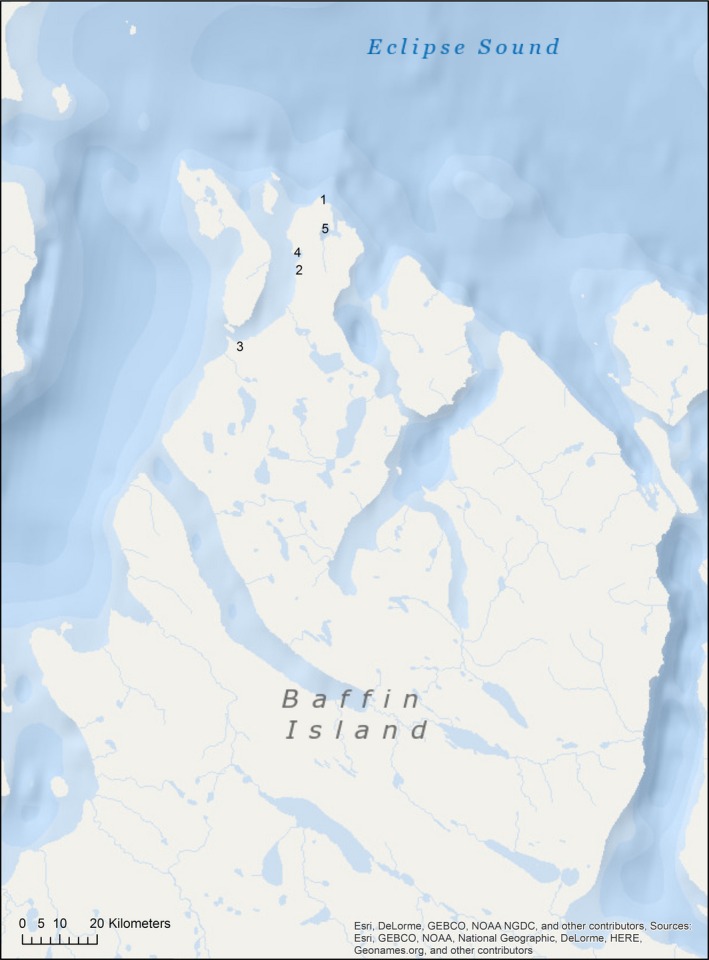
Study area: (1) Cape Hatt, (2) the outer side of Bay 11/12, (3) the south of Ragged Island, (4) Bay 11/12, (5) Z lagoon.

All study sites were located at and around Cape Hatt and Ragged Channel in the northwest of Baffin Island, Nunavut, Canada (Fig. 1), which were visited from August 17 until September 4, 2009. These included exposed sites, specifically at Cape Hatt (72°30′ N, 79°47′ W), the outer side of Bay 11/12 as defined in the context of the BIOS project (72°27′ N, 79°50′ W; Cross et al. 1987, Sempels 1987), the south of Ragged Island (72°24′ N, 79°59′ W and 72°24′ N, 80°00′ W), and more sheltered sites, specifically the north shore (72°28′ N, 79°50′ W) and south shore (72°27′ N, 79°50′ W) of Bay 11/12, and Z lagoon (72°29′ N, 79°46′ W and 72°28′ N, 79°45′ W, respectively). Diving operations followed the principles of the UK Diving at Work Regulations so far as was reasonably practicable (Sayer 2004). A total of 50 diving operations were performed at the aforementioned sites, with an average maximum operating depth of 10.4 m and a depth limit of 15 m as imposed by the terms of the expedition insurance, which was influenced by the lack of suitable recompression facilities in the region (Sayer et al. 2013). Details of how diving operations were conducted safely in this extremely remote location were described in Sayer et al. (2013). Underwater photographs and video footage were taken with two digital SLR still cameras Nikon D‐300(Nikon Co. Ltd., Tokyo, Japan) and Canon EOS 5D (Canon Inc., Tokyo, Japan) and a high‐definition video camera Sony HDR‐HC7 (Sony, Tokyo, Japan), all in underwater housings.

During the dives, qualitative surveys of benthic habitats and their macroscopic seaweed diversity were conducted, usually from the shore to the maximum depth accessible with two divers tethered on a single 100 m long L‐lifeline with a maximum operational depth of 15 m, as limited by insurance terms (whichever was reached first; Sayer et al. 2013): seaweed specimens were collected haphazardly as entire thalli, where feasible at least in triplicate, which were subsequently conserved as herbarium specimens on Bristol paper or (for the smaller specimens, <1 cm) on microscope slides using acetocarmine as fixative and dye and 50% Karo^™^ syrup as embedding medium (Müller and Ramírez 1994). Where appropriate, algal specimens were investigated in the field with a small compound microscope with 25×, 100× and 750× magnification. During the dives and where appropriate (e.g., to assess coverage or canopy composition), biomass estimates were made by visual assessment in rough classes (no vegetation, partial cover, or complete cover, respectively – not shown).

Diving also enabled the collection of sediment and benthic substratum samples in sterile 15 or 50 mL Falcon^™^ tubes. Samples were collected between 21 and 30 August 2009 in order to cover all types of substratum occurring in the study region. Unialgal laboratory cultures from such samples were established following the Germling Emergence Method (Peters et al. 2015), which involved placing part or all of a substratum sample in a petri dish with half‐strength Provasoli‐enriched sea water (Starr and Zeikus 1987) at around 4°C with a 12:12 h photoperiod.

The intertidal and supralittoral were surveyed on foot at all sites were dives were surveyed in parallel to the dives, typically covering a minimum of 50 m of shore line in both directions from the entry point of each dive.

Unialgal isolates obtained from incubated substrata were further characterized by sequencing of the DNA barcode locus COI (cox1) and cox3. DNA extractions employed the DNeasy Plant Mini Kit (Qiagen, Hilden, Germany). Polymerase chain reactions (PCR) were performed using primer pairs for partial mitochondrial cox1 (COI; both 5P and 3P) and cox3‐5P gene regions (Table 1). PCR amplifications were performed in a total volume of 25 μL, containing 1.25 units · μL^−1^ of Taq DNA Polymerase (Promega, Madison, WI, USA), 1× GoTaq^™^ buffer, 5 mM MgCl_2_, 1.25 mM dNTPs, 1.87 mM of each primer and 1 μL of template DNA (5–50 ng · μL^−1^). PCR amplification for the primers pair COI‐789F/COI‐1378R was carried out with an initial denaturation at 94°C for 5 min; followed by 40 cycles of amplification consisting of denaturation at 94°C for 30 s, annealing at 46°C for 30 s and elongation at 72°C for 40 s. The 40 cycles were followed by a final extension at 72°C for 5 min. For the cox1‐GazF1/cox1‐GazR1 PCR amplification was carried out with an initial denaturation at 94°C for 2 min; followed by 35 cycles of amplification consisting of denaturation at 94°C for 30 s, annealing at 50°C for 30 s and elongation at 72°C for 1 min. The 35 cycles were followed by a final extension at 72°C for 5 min.

**Table 1:** Oligonucleotide primers used for PCR amplification and sequencing

Locus	Primers	Primer sequences	Reference
5′‐cox1 (COI)	117F	TTTCHACNAAYCAYAAAGATAT	Bittner et al. (2008)
	784R	ACTTCDGGRTGDCCAAAAAACCA	Bittner et al. (2008)
5′‐cox1 (COI)	gazF2	CCAACCAYAAAGATATWGGTAC	Saunders (2005)
	gazR2	GGATGACCAAARAACCAAAA	Lane et al. (2007)
3′‐cox1	789R	TNTAYCARCATTTATTTTGGTT	Silberfeld et al. (2010)
	1378R	TCYGGNATACGNCGNGGCATACC	Silberfeld et al. (2010)
cox3	44F	CAACGNCAYCCWTTTCATTT	Silberfeld et al. (2010)
	551R	TGCATASCNGTRAADGCWAYRGC	This study
	739R	CATCNACAAAATGCCAATACCA	Silberfeld et al. (2010)
	67F	TTRGTTGAYCCNAGYCCNTGGC	Silberfeld et al. (2010)
	623R	CATGAAANCCATGRAANCCNGTAG	Silberfeld et al. (2010)

PCR products were run on a GelRed^™^ (Biotium, Hayward, CA ) TBE agarose (1.2%) gel to check for amplification and correct length. A single reaction product of ~50 ng DNA was purified using the QIAquick PCR Purification Kit (Qiagen) and sequenced on both strands by the Source Bioscience sequencing service using the same primers as employed for PCR.

The alignment of each gene consensus sequence was created with BioEdit Sequence Alignment Editor (Hall 1999) and then the sequences were compared to published data by means of NCBI BLAST searches (Altschul et al. 1997). Sequence alignments and Neighbor‐Joining distance analyses were made as in Peters et al. (2015).

Newly generated sequences were deposited in the European Nucleotide Archive, accessions LT546264 to LT546319.

Strains identified at least to genus have been deposited in the Culture Collection of Algae and Protozoa (CCAP).

## Results

Algal records for this region were made during four expeditions led by RTW and FCK, now spanning 25 years (1984–2009). A total of 33 Phaeophyceae, 24 Rhodophyceae, 2 Chlorophyceae, 12 Ulvophyceae, 1 Trebouxiophyceae, 1 Dinophyceae, and one cyanobacterium were recorded (Table 2), together with the oomycete pathogen *E. dicksonii*.

**Table 2:** Species checklist

Cyanobacteria	
*Calothrix scopulorum* C. Agardh	1982, 2004*, 2009**
Chlorophyta	
Chlorophyceae	
*Chlorochytrium dermatocolax* Reinke	1982, 2004*, 2009**
*Chlorochytrium schmitzii* Rosenvinge	1982, 2004*
Ulvophyceae	
*Acrosiphonia arcta* (Dillwyn) Gain	1982, 2004*, 2009**
*Ochlochaete hystrix* Thwaites ex Harvey	1982, 2004*
*Blidingia minima* (Nägeli ex Kützing) Kylin	1982, 2004*, 2009**
*Chaetomorpha melagonium* (F. Weber et D. Mohr) Kützing	1982, 2004*, 2009**
*Chaetomorpha linum* (O. F. Müller) Kützing	1982, 2004*
*Pseudendoclonium submarinum* Wille	1982, 2004*, 2009**
*Rhizoclonium riparium* (Roth) Harvey	1982, 2004*
*Spongomorpha aeruginosa* (L.) Hoek	1982, 2004*
*Ulva prolifera* O. F. Müller	1982, 2004*
*Ulva rigida* C. Agardh	1982, 2004*, 2009**
*Ulothrix implexa* (Kützing) Kützing	1982, 2004*
*Urospora wormskioldii* (Mertens *ex* Hornemann) Rosenvinge	1982, 2004*
Trebouxiophyceae	
*Stichococcus bacillaris* Nägeli	1982*
Ochrophyta	
Phaeophyceae	
*Agarum clathratum* Dumortier	1982, 2004*, 2009**#
*Alaria esculenta* (L.) Greville	1982, 2004*, 2009**
*Battersia arctica* (Harvey) Draisma, Prud'homme & H. Kawai	1982, 2004**, 2009**
*Chaetopteris plumosa* (Lyngbye) Kützing	1982, 2004*, 2009**
*Chorda filum* (L.) Stackhouse	1982*
*Coelocladia arctica* Rosenvinge	1982, 2004*
*Delamarea attenuata* (Kjellman) Rosenvinge	1982*
*Desmarestia aculeata* (L.) J.V. Lamouroux	1982, 2004*, 2009**#
*Desmarestia viridis* (O.F. Müller) J.V. Lamouroux	1982, 2004*
*Desmarestia* sp.	2009#
*Dictyosiphon foeniculaceus* (Hudson) Greville	1982, 2004*, 2009**
*Dictyosiphon* sp.	2009#
*Ectocarpus siliculosus* (Dillwyn) Lyngbye	1982, 2004*, 2009**
*Elachista fucicola*	1982, 2004*, 2009**
*Fucus evanescens* C. Agardh	1982, 2004*, 2009**
*Halosiphon tomentosus* (Lyngbye) Jaasund	1982, 2004*, 2009**
*Hincksia* sp.	2009#
*Laminaria solidungula* J. Agardh	1981, 2004*, 2009**
*Laminariocolax aecidioides* (Rosenvinve) A.F. Peters	2009#
*Leptonematella fasciculata* (Reinke) P.C. Silva	1982, 2004*, 2009**
*Omphalophyllum ulvaceum* Rosenvinge	1982*
*Petroderma maculiforme* (Wollny) Kuckuck	1982, 2004*, 2009**
*Phaeostroma longisetum* (Lund) Pedersen	1982, 2004*, 2009**
*Platysiphon glacialis* (Rosenvinge) H. Kawai et T. Hanyuda	1982, 2004*, 2009**
*Punctaria tenuissima* (C. Agardh) Greville	1982, 2004*, 2009**
*Pylaiella littoralis* (L.) Kjellman	2009#
*Pylaiella washingtoniensis* C.C. Jao	2009**#
*Saccharina latissima* (L.) Lane, Mayes, Druehl et Saunders	1982, 2004*, 2009**#
*Saccharina longicruris* (Bachelot de la Pylaie) Kuntze	1982, 2004*, 2009**
*Scytosiphon lomentaria* (Lyngbye) J. Agardh	2009**
*Sphaceloderma caespitula* (Lyngbye) Draisma, Prud'homme van Reine et Kawai	1982*
*Stragularia clavata* (Harvey) Hamel	1982, 2004*, 2009**
Dinophyceae (1)	
*Rufusiella foslieana* (Hansgirg) T. Christensen	1982, 2004*
Rhodophyta	
*Ahnfeltia plicata* (Hudson) Fries	1982, 2004*
*Clathromorphum compactum* (Kjellman) Foslie	1982, 2004*
*Ceratocolax hartzii* Rosenvinge	1982, 2004*
*Coccotylus truncatus* (Pallas) M.J. Wynne et J.N. Heine	1982*
*Devaleraea ramentacea* (L.) Guiry	1982, 2004*, 2009**
*Dilsea socialis* (Postels & Ruprecht) Perestenko	1982, 2004*, 2009**
*Fimbrifolium dichotomum* (Lepechin) G.I. Hansen	1982*, 2009**
*Harveyella mirabilis* (Reinsch) F. Schmitz & Reinke	1982, 2004*
*Halosacciocolax kjellmanii* S. Lund	1982, 2004*
*Leptophytum foecundum* (Kjellman) W.H. Adey	1982, 2004*
*Leptophytum tenue* (Kjellman) Athanasiadis & W.H. Adey	1982, 2004*
*Lithothamnion glaciale* Kjellman	1982, 2004, 2009
*Odonthalia dentata* (L.) Lyngbye	1982*, 2004**
*Palmaria palmata* (L.) F. Weber & D. Mohr	1982, 2004*, 2009**
*Pantoneura baerii* (Ruprecht) Kylin	1982*
*Phycodrys rubens* (L.) Batters	1982, 2004*
*Polysiphonia arctica* J. Agardh	1982*, 2009
*Ptilota serrata* Kützing	1982*
*Rhodochorton purpureum* (Lightfoot) Rosenvinge	1982, 2004*, 2009**
*Rhodomela confervoides* (Hudson) P.C. Silva	1982, 2004*, 2009**
*Rhodomela lycopodioides* (L.) C. Agardh	1982, 2004*
*Rhodophysema elegans* (P.L. Crouan et H.M. Crouan *ex* J. Agardh) P.S. Dixon	1982*
*Scagelia pylaisaei* (Montagne) M.J. Wynne	1982, 2004*, 2009**
*Turnerella pennyi* (Harvey) F. Schmitz	1982, 2004*, 2009**

All 1981–1983 records are from Cross et al. (1987). Previously unpublished records by R.T. Wilce are marked with one asterisk (*). Records based on macroscopic collections from the 2009 expedition are marked with two asterisks (**), while records based upon the Germling Emergence Method in combination with DNA barcoding within the framework are marked with a hashtag (#).

### Environmental conditions

The period of the 2009 expedition was marked by a lack of sea ice or even remnants of it. Use of dive computers allowed for a rough assessment of temperature regimes at dive sites. In the sheltered water body of Z Lagoon, we recorded surface temperatures as high as 7°C. Beneath a thermocline (during all dives, between 4 and 7 m depth), the temperature decreased to ~2°C, which was also the temperature typically encountered at high‐current, exposed open‐water sites dived during this expedition such as Cape Hatt and Ragged Island. The only ice in the sea observed during the entire expedition were icebergs, which according to the local population had reportedly drifted to Eclipse Sound and Ragged Channel from ice shelves in Greenland.

### Supralittoral

Few macroalgae were encountered in the supralittoral. Most apparent was the cyanobacterium *Calothrix scopulorum* as a characteristic black crust immediately below an upper littoral barren zone. From previous studies (Wilce, unpublished) of the North Baffin littoral more than 30 species are known to occur in crevices and pools. Several algal species are macroscopic, e.g., *Rhodochorton purpureum*,* Blidingia minima*,* Ulva clathrata*,* U. prolifera* and a host of smaller stature species. Most are diminutive, in localized protected niches, and virtually all require microscopic study for their identification.

### Intertidal habitats

Tides on the coasts of northern Baffin Island are predominantly semi‐diurnal (Buckley et al. 1987, Canadian Hydrographic Services 2016). The maximum tidal range in the Eclipse Sound area is ~2.5 m (Buckley et al. 1987), while it is around 2.2 m at nearby Pisiktarfik Island (Canadian Hydrographic Services 2016). Generally, the intertidal at all sites surveyed around Cape Hatt was barren and only sparsely populated by macroalgae. Occasionally, *R. purpureum* was found in sheltered microhabitats between and beneath boulders. Only at one site, a small stand of *Fucus evanescens* and *Scytosiphon lomentaria* occurred on a rocky platform – obviously this location was spared from the severe impacts of ice scouring, which is a major abiotic factor impacting benthic communities in polar regions (e.g., Conlan et al. 1998, Barnes 1999, Conlan and Kvitek 2005). Tidal pools were colonized by summer annuals, especially *Pylaiella* sp. and *B. minima*.

### Seaweed diversity of benthic subtidal habitats

#### Exposed environments

**Figure 2:**
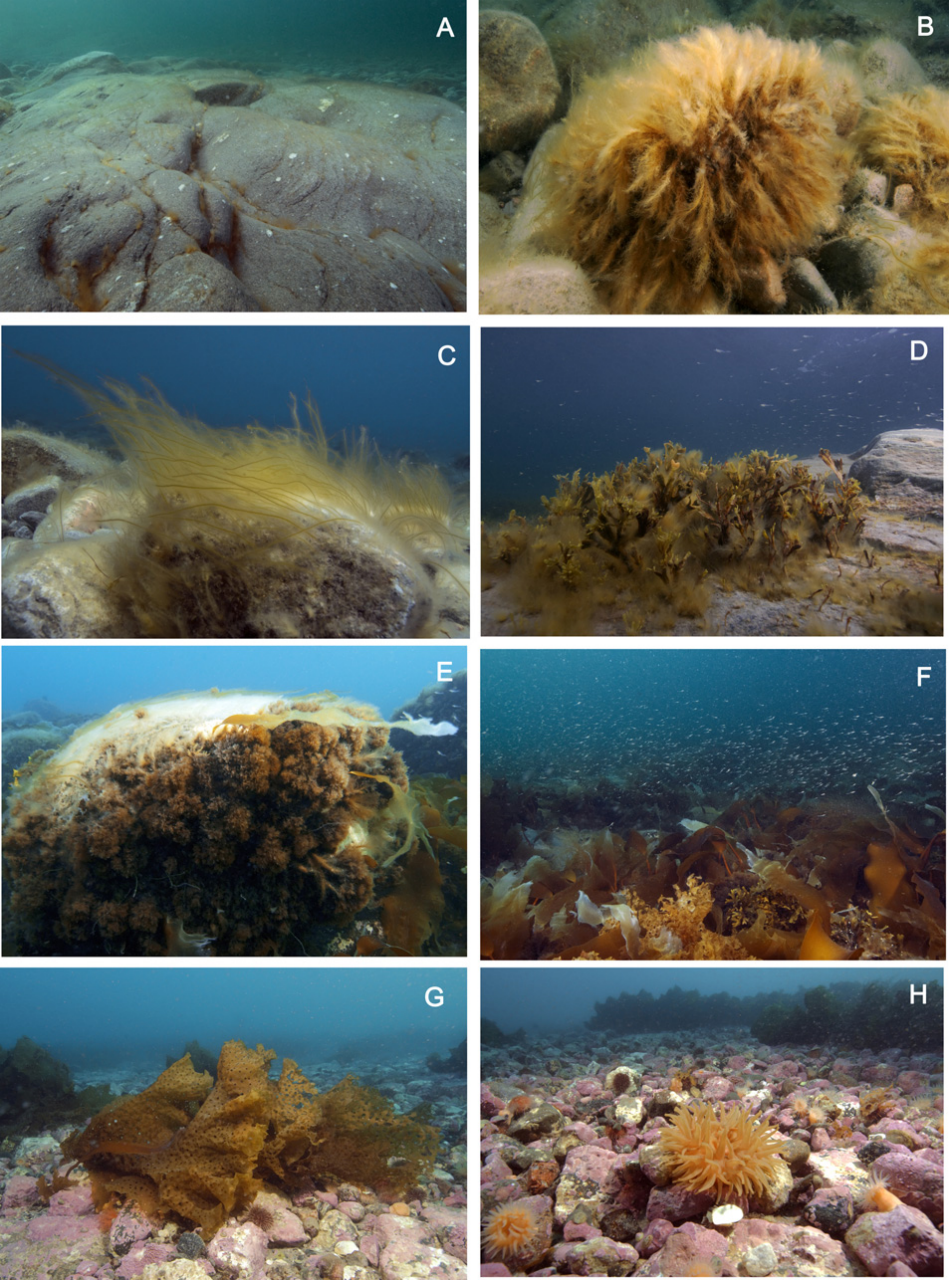
In situ photographs of the phytobenthos of northern Baffin Island at exposed locations, taken during the 2009 expedition to northern Baffin Island. (A) Barren rocks, ~1 m below low water – only *Pylaiella* as a summer annual is visible in crevices. (B) Epilithic *Pylaiella*, ~2 m deep. (C) *Halosiphon tomentosus*, a typical representative of the upper sublittoral at ~3 m depth. (D) *Fucus evanescens* in the sublittoral at ~3 m depth. (E) Rocks at ~3 m depth, demonstrating the effects of ice scouring – the upper side of the rocks, which would harbor rich macroalgal vegetation anywhere else in the world outside the polar regions, is free of vegetation cover. (F) Dense canopy of the kelps *Saccharina latissima*,* S. longicruris*,* Laminaria solidungula* and *Alaria esculenta* at ~7 m depth. (G) *Agarum clathratum* is typical of the lower sublittoral vegetation at ~10–15 m depth. (H) Once the kelp forest becomes patchy and ultimately disappears at ~15 m depth, coralline red algae dominate hard substrata.

Few perennial macroalgae occurred on both rocky surfaces and pebbles within the first 3 m below low tide level (Fig. 2, A–C). Typical taxa encountered here included epilithic *Pylaiella* sp., *Halosiphon tomentosus* (Fig. 2C), *Stictyosiphon tortilis,* and *Dictyosiphon* sp. (Fig. 2, A and B).

*Fucus evanescens* (Fig. 2D) occurred mostly at and beneath 3 m depth, often with *Pylaiella* sp. and *Elachista* sp. as epiphytes – contrasting with its occurrence in the low intertidal and upper subtidal in boreal/cold‐temperate environments (Schueller and Peters 1994) – but it could be found closer to the surface (or rarely in the intertidal, see above) in ice‐sheltered locations. The impact of sea ice on phytobenthic communities was severe, leaving the upper parts of rocks (above around 3 m depth) devoid of perennial vegetation (Fig. 2E). Iceberg scouring was intense, often leaving characteristic traces up to a meter deep and from a few meters to several hundred meters in length, usually devoid of any vegetation of perennial algae.

Kelps (*Laminaria solidungula*,* Saccharina latissima,* and *Alaria esculenta*), with thalli up to several meters in length, had their upper limit at around 5 m depth and occurred down to 10–12 m, often in a mixed canopy together with *Desmarestia aculeata* and the red alga *Dilsea socialis*. While the non‐acid producing *D. aculeata* was observed to be a major canopy‐forming species in this community (Fig. 2F), acid‐producing *Desmarestia* species (Yang et al. 2014) were much smaller both in terms of thallus size and biomass contribution to this community. Sporophytes of *D. viridis* were collected at Cape Hatt in 1981 and 2004, but not during the 2009 expedition. Gametophytes of a closely related, so far unknown *Desmarestia* species were isolated from substratum samples collected in 2009 (see below).

The characteristic kelp species occurring mostly beneath 10 m was *Agarum clathratum*. Under the canopy of this kelp and in open patches, the seabed was dominated by coralline red algae, especially *Leptophytum foecundum* and *Lithothamnion glaciale*.

**Figure 3:**
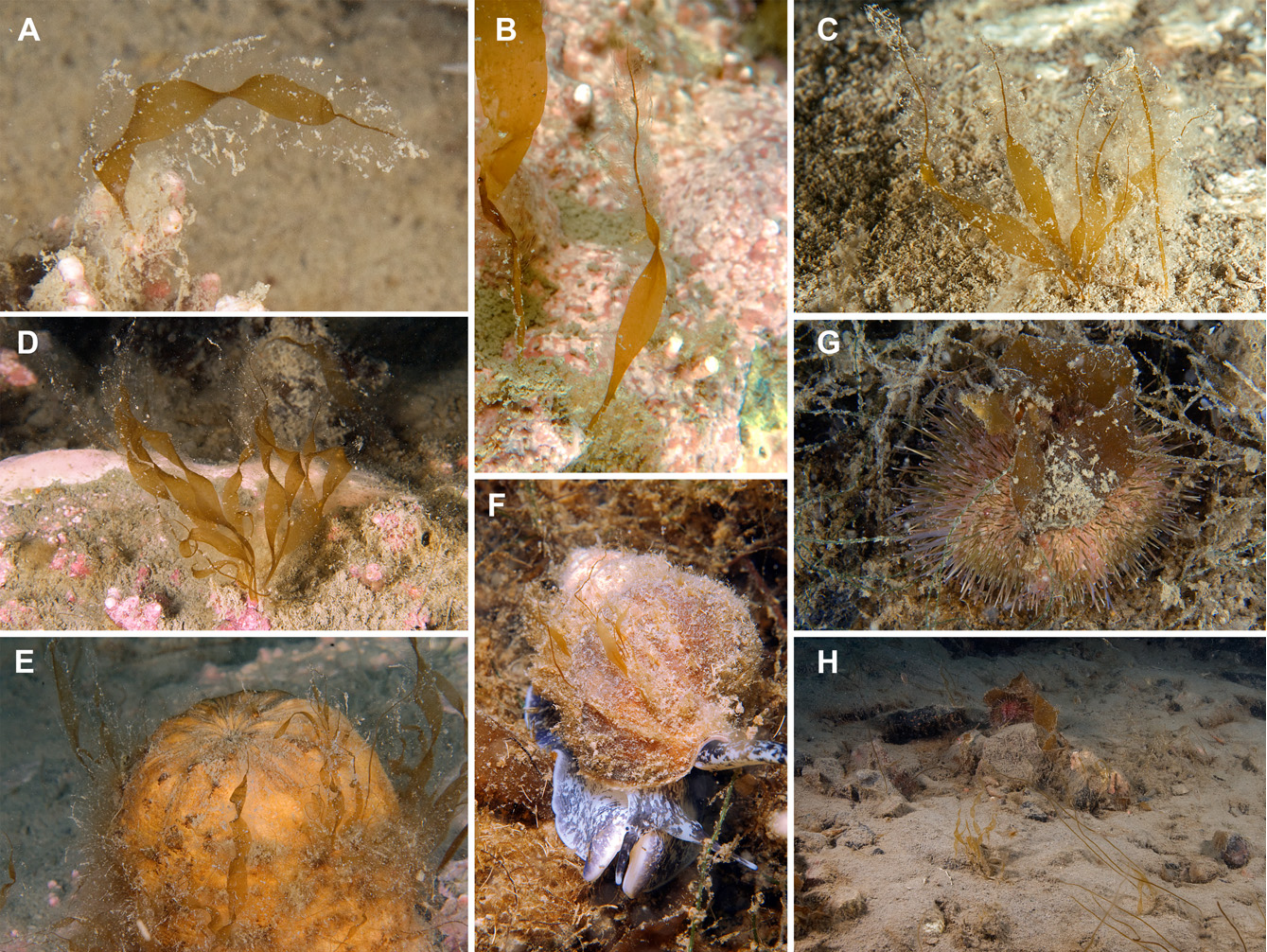
Underwater images of *Platysiphon glacialis*, taken off Cape Hatt in the Ragged Channel area of northern Baffin Island. *Platysiphon* can attach to a remarkable diversity of substrata, including rock, sea shells, coralline algae, marine snails, and sponges (A–F). The perennial form (previously denominated *Punctaria glacialis*, G and H) lives often detached and is commonly found on the back of sea urchins. (H) It shows a typical habitat of *Platysiphon* at ~10 m depth. *Platysiphon* is frequently found together with *Halosiphon tomentosus* (C–H).

The brown alga *Platysiphon glacialis* (Kawai et al. 2015a,b) was typically encountered beneath 3 m depth. In situ observations of this taxon are reported here (Fig. 3). This Arctic endemic was observed on a wide range of substrata from ~3 to 12 m depth, including sea shells, snails, pebbles, solid rocky substrates, and also epiphytic on kelps (especially *A. clathratum*).

Beneath 12 m, the kelp stands were more scattered and sparse, with *A. clathratum* being the only species occurring here (Fig. 2G). At ~15 m depth and on solid substrata, coralligenous communities (especially *L. foecundum* (Kjellman) Adey and *L. glaciale*) completely dominated the phytobenthos (Fig. 2H).

#### Sheltered water bodies‐ Fjords and bays

**Figure 4:**
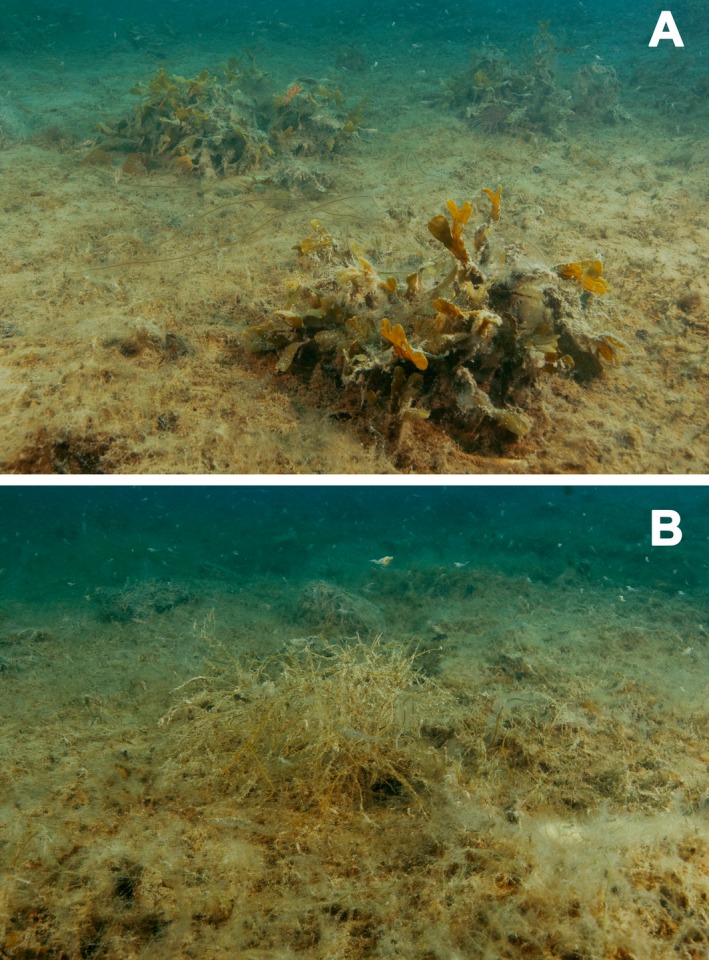
Fjordic environments with low currents are characterized by masses of dead macrophyte biomass, covered by a layer of live filamentous algae and, occasionally, *Platysiphon glacialis*,* Fucus evanescens*,* Saccharina latissima*,* S. longicruris*,* Laminaria solidungula,* and *Desmarestia aculeata*. Typically, everything is covered with large amounts of fine sediment.

The species composition of these environments was different from exposed benthic habitats (Fig. 4). Both *L. solidungula* and *S. latissima* were common, but *A. esculenta* and *A. clathratum* were mostly absent. Kelps and mollusc shells often constituted substata for *P. glacialis*. The colonial, tubular diatom *Berkeleya rutilans* was always present, but less common than the dominant macroalgal species. Taxa of small stature, epiphytes and endophytes were numerous with up to 30 additional species as minor associates. Beneath ~5 m, the interior of such sheltered environments was covered by a loose‐lying algal mass consisting of *Pylaiella*,* Dictyosiphon,* and *Stictyosiphon*, covering an often dense, thick layer of organic matter that became anoxic within a few centimetres depth (lack of oxygen was visible due to the black color of the fine particles present in these layers, demonstrating the occurrence of sulfate reduction).

### Algal isolates from substratum samples

From a total of 23 substratum samples, 66 clonal algal cultures were obtained, six of which were diatoms (not shown), one red, seven green, and 52 brown algae. Sequences were obtained for 48 of the latter. Pruning of putative culture duplicates (i.e., clones from the same substratum sample showing identical sequences) left 34 clones. Of these, 5′‐COI sequences (658 bp) were obtained for 26, 3′‐COI (561 bp) for 16, and cox3‐5P (694 bp) for 25 clones (Table 3).

**Table 3:** BLAST results. High genetic similarities (97%–100%) to published sequences in bold face. CCAP: Culture Collection of Algae and Protozoa

Marker	CCAP accession	Clone	Similarity	BLAST	Species
5′‐COI		BI003	**1.00**	JX571962	*Pylaiella washingtoniensis*
5′‐COI	CCAP 1322/2	BI008	**0.99**	LM995048	*Laminariocolax aecidioides*
5′‐COI	CCAP 1330/10	BI022	0.88	LM995398	*Acinetosporaceae* sp. *4 AP‐2014*
5′‐COI		BI023	**1.00**	JX571962	*Pylaiella washingtoniensis*
5′‐COI		BI024	**0.98**	JX571962	*Pylaiella washingtoniensis*
5′‐COI		BI027	**0.99**	LM994982	*Laminariocolax macrocystis*
5′‐COI		BI029	0.89	LM994993	*Microspongium alariae*
5′‐COI		BI030	0.89	LM995296	*Chordariaceae* sp. *15 AP‐2014*
5′‐COI		BI032	**0.99**	LM994982	*Laminariocolax macrocystis*
5′‐COI		BI033	0.88	LM995406	*Acinetosporaceae* sp*. 3 AP‐2014*
5′‐COI		BI038	**1.00**	JX571962	*Pylaiella washingtoniensis*
5′‐COI		BI039	0.92	LM995092	*Chordariaceae* sp. *10 AP‐2014*
5′‐COI		BI040	0.88	LM995406	*Acinetosporaceae* sp. *3 AP‐2014*
5′‐COI		BI042	0.89	LM994993	*Microspongium alariae*
5′‐COI		BI043	0.91	LM995296	*Chordariaceae* sp. *15 AP‐2014*
5′‐COI		BI044	0.89	LM995296	*Chordariaceae* sp*. 15 AP‐2014*
5′‐COI		BI047	**0.99**	JX571962	*Pylaiella washingtoniensis*
5′‐COI		BI048	0.93	LM995297	*Chordariaceae* sp*. 16 AP‐2014*
5′‐COI	CCAP 1316/1	BI051	**0.99**	JX572013	*Dictyosiphon* sp. *1GWS*
5′‐COI		BI059	0.89	LM995208	*Hincksia hincksiae*
5′‐COI	CCAP 1306/45	BI061	**1.00**	HE866759	*Desmarestia aculeata*
5′‐COI	CCAP 1300/1	BI062	**1.00**	KJ960265	*Agarum clathratum*
5′‐COI		BI063	**1.00**	FJ409199	*Saccharina latissima*
5′‐COI		BI064	0.95	AY500367	*Desmarestia viridis*
5′‐COI	CCAP 1306/46	BI065	0.95	AY500367	*D. viridis*
5′‐COI	CCAP 1318/1	BI077	**0.99**	GU097790	*S. latissima*
3′‐COI		BI003	**0.98**	AB899179	*Pylaiella* sp.
3′‐COI	CCAP 1333/1	BI011	0.91	EU681415	*Petalonia fascia*
3′‐COI		BI027	0.94	GQ368263	*Hydroclathrus clathratus*
3′‐COI		BI032	0.94	GQ368263	*Hydroclathrus clathratus*
3′‐COI		BI033	0.88	AB899179	*Pylaiella* sp.
3′‐COI		BI037	0.91	GQ368263	*Hydroclathrus clathratus*
3′‐COI		BI039	0.90	GQ368263	*Hydroclathrus clathratus*
3′‐COI		BI040	0.88	AB899179	*Pylaiella* sp.
3′‐COI		BI041	0.94	GQ368263	*Hydroclathrus clathratus*
3′‐COI		BI045	0.91	AB775232	*Stictyosiphon soriferus*
3′‐COI	CCAP 1334/1	BI052	0.88	JF796540	*Ectocarpus fasciculatus*
3′‐COI		BI059	0.91	EU681410	*Hincksia granulosa*
3′‐COI	CCAP 1306/45	BI061	**0.99**	EU681402	*D. aculeata*
3′‐COI	CCAP 1300/1	BI062	**0.97**	GQ368254	*A. clathratum*
3′‐COI		BI063	**0.99**	KM675818	*S. latissima*
3′‐COI		BI064	0.95	NC007684	*D. viridis*
cox3‐5P		BI003	**0.98**	AB526446	*Pylaiella* sp.
cox3‐5P	CCAP1322/2	BI008	0.91	JF796553	*Chordaria flagelliformis*
cox3‐5P	CCAP 1333/1	BI011	0.93	KF700318	*Petalonia zosterifolia*
cox3‐5P	CCAP 1330/9	BI019	**0.99**	AJ277126	*Pylaiella littoralis*
cox3‐5P		BI022	0.87	EU681451	*Hicksia granulosa*
cox3‐5P	CCAP 1330/10	BI023	**0.98**	AB526446	*Pylaiella* sp.
cox3‐5P		BI024	**0.97**	AB526446	*Pylaiella* sp.
cox3‐5P		BI029	0.88	EU681459	*Punctaria latifolia*
cox3‐5P		BI030	0.88	FP885846	*Ectocarpus siliculosus*
cox3‐5P		BI037	0.91	JF796553	*Chordaria flagelliformis*
cox3‐5P		BI038	**0.99**	AB526446	*Pylaiella* sp.
cox3‐5P		BI042	0.88	EU681459	*Punctaria latifolia*
cox3‐5P		BI043	0.87	EU681459	*Punctaria latifolia*
cox3‐5P		BI044	0.88	FP885846	*Ectocarpus siliculosus*
cox3‐5P		BI047	**0.98**	AB526446	*Pylaiella* sp.
cox3‐5P		BI048	0.92	JF796553	*Chordaria flagelliformis*
cox3‐5P	CCAP 1316/1	BI051	0.94	JF796554	*Dictyosiphon foeniculaceus*
cox3‐5P	CCAP 1334/1	BI052	0.85	FP885846	*Ectocarpus siliculosus*
cox3‐5P		BI059	0.87	EU681451	*Hicksia granulosa*
cox3‐5P		BI063	**0.99**	KM675818	*S. latissima*
cox3‐5P		BI064	0.96	AY500367	*D. viridis*
cox3‐5P		BI065	0.96	AY500367	*D. viridis*
cox3‐5P		BI067	0.85	JF796554	*Dictyosiphon foeniculaceus*
cox3‐5P		BI068	0.85	JF796554	*Dictyosiphon foeniculaceus*
cox3‐5P	CCAP 1318/1	BI077	**0.99**	KM675818	*S. latissima*

**Figure 5:**
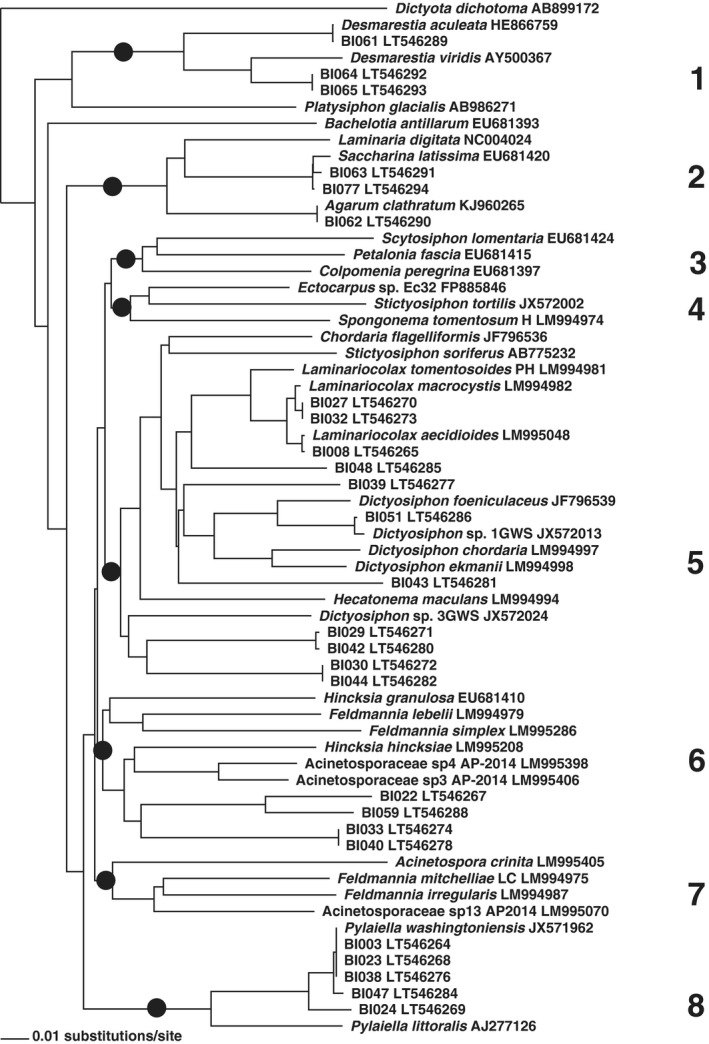
Neighbor‐Joining phylogram displaying 5′‐COI clustering of 63 brown algal sequences, including 37 public references and sequences of 26 clonal cultures raised from environmental samples collected at Baffin Island (taxon names starting with BI). The eight major clusters obtained, in part corresponding to known higher taxa, are numbered consecutively 1–8 to the right of the tree, their roots are indicated by dark circles. 1. Desmarestiales, 2. Laminariales, 3. Scytosiphonaceae, 4. Ectocarpaceae, 5. Chordariaceae, 6. *Hincksia* cluster, 7. *Acinetospora* cluster, 8. *Pylaiella* cluster. Clusters 6–8 are traditionally classified together in Acinetosporaceae, however, they did not form a single clade in our analyses.

**Figure 6:**
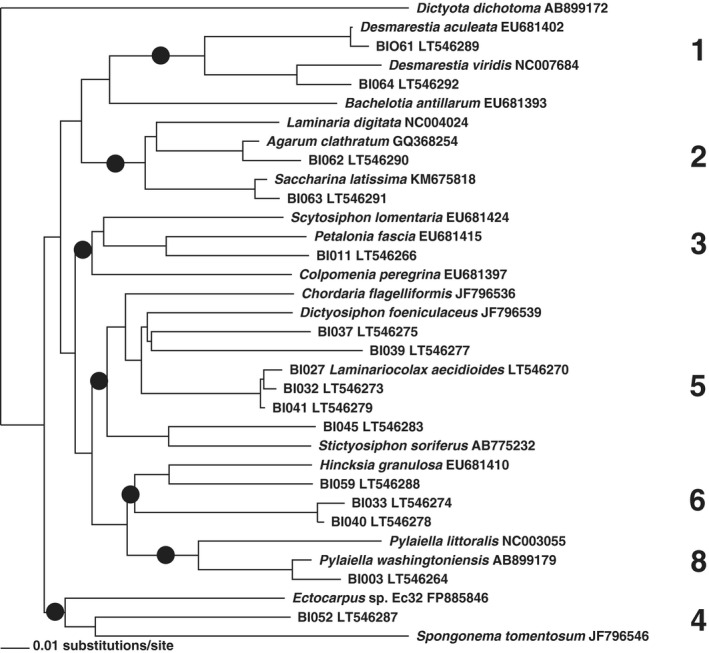
Neighbor‐Joining phylogram displaying 3′‐COI clustering of 35 brown algal sequences, including 19 public references and sequences of 16 clonal cultures raised from environmental samples collected at Baffin Island (taxon names starting with BI). The major clusters obtained are provided as numbers, corresponding to the clusters in Figure 5. Cluster 7 is absent because of unavailable reference sequences.

**Figure 7:**
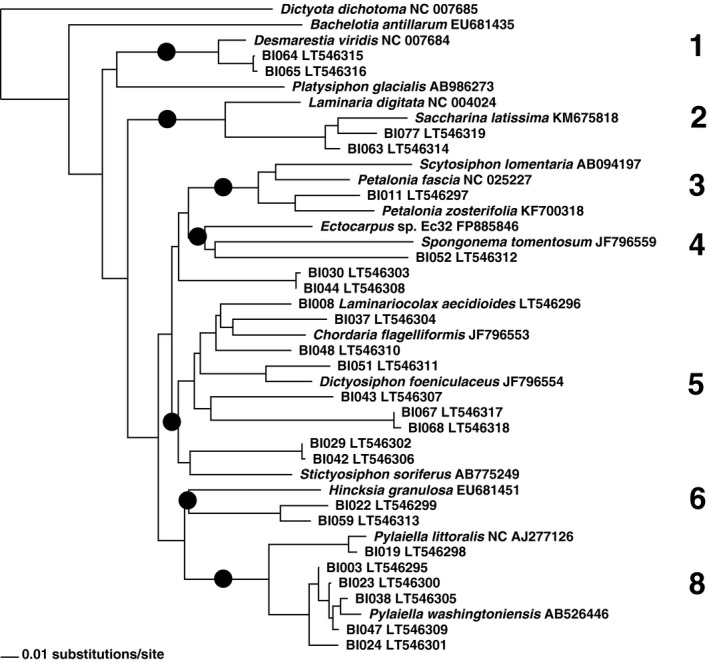
Neighbor‐Joining phylogram displaying cox3 clustering of 42 brown algal sequences, including 17 public references and sequences of 25 clonal cultures raised from environmental samples collected at Baffin Island (designations starting with BI). The major clusters obtained are provided as numbers, corresponding to the clusters in Figure 5. Cluster 7 is absent because of unavailable reference sequences.

Neighbor‐joining distance analyses of the data and added reference sequences resulted in grouping of the sequences in eight clusters corresponding to recognized higher taxa (Figs. 5, 6, 7). A majority of 28 clones grouped with reference taxa of the Ectocarpales sensu lato, three with Laminariales and three with Desmarestiales. Applying a species‐level cut‐off of ~2% genetic divergence, the 34 clones were broken down to 20 species (15 revealed by 5′‐COI, 4 more by 3′‐COI, and one more by 5′‐cox3; Figs. 4, 5, 6; Table 4).

**Table 4:** Clonal cultures obtained by germling emergence from substratum collected at Baffin Island, and their identification by means of molecular barcoding. Strains identified to species in bold face

Code	Total evidence identity	5′‐COI identity	3′‐COI identity	cox3‐5P identity	Sequence accessions	Comment or name in sequence data bases
BI003	***Pylaiella washingtoniensis***	*Pylaiella washingtoniensis*	*Pylaiella*, not *littoralis*	*Pylaiella*, not *littoralis*	LT546264, LT546295	
BI008	***Laminariocolax aecidioides***	*Laminariocolax aecidioides*		Chordariaceae	LT546265, LT546296	
BI011	Scytosiphonaceae		Scytosiphonaceae	Scytosiphonaceae	LT546266, LT546297	*Petalonia* sp. 1 AP‐2016
BI019	***Pylaiella littoralis***			*Pylaiella littoralis*	LT546298	
BI022	*Hincksia* cluster	*Hincksia* cluster		*Hincksia* cluster	LT546267, LT546299	Acinetosporaceae sp. 1 AP‐2016
BI023	***Pylaiella washingtoniensis***	*Pylaiella washingtoniensis*		*Pylaiella*, not *littoralis*	LT546268, LT546300	
BI024	***Pylaiella*** **, not** ***littoralis***	*Pylaiella*, not *littoralis*		*Pylaiella*, not *littoralis*	LT546269, LT546301	Closely related to *P. washingtoniensis*
BI027	***Laminariocolax aecidioides***	*Laminariocolax aecidioides*	Chordariaceae		LT546270	
BI029	Chordariaceae	Chordariaceae		Chordariaceae	LT546271, LT546302	Chordariaceae sp. 1 AP‐2016
BI030	Chordariaceae	Chordariaceae		no clear affiliation	LT546272, LT546303	Chordariaceae sp. 2 AP‐2016
BI032	***Laminariocolax aecidioides***	*Laminariocolax aecidioides*	Chordariaceae		LT546273	
BI033	*Hincksia* cluster	*Hincksia* cluster	*Hincksia* cluster		LT546274	Acinetosporaceae sp. 2 AP‐2016
BI037	Chordariaceae		Chordariaceae	Chordariaceae	LT546275, LT546304	Chordariaceae sp. 3 AP‐2016
BI038	***Pylaiella washingtoniensis***	*Pylaiella washingtoniensis*		*Pylaiella*, not *littoralis*	LT546276, LT546305	
BI039	Chordariaceae	Chordariaceae	Chordariaceae		LT546277	Chordariaceae sp. 4 AP‐2016
BI040	*Hincksia* cluster	*Hincksia* cluster	*Hincksia* cluster		LT546278	Acinetosporaceae sp. 2 AP‐2016
BI041	***Laminariocolax aecidioides***		Chordariaceae		LT546279	
BI042	Chordariaceae	Chordariaceae		Chordariaceae	LT546280, LT546306	Chordariaceae sp. 1 AP‐2016
BI043	Chordariaceae	Chordariaceae		Chordariaceae	LT546281, LT546307	Chordariaceae sp. 5 AP‐2016
BI044	Chordariaceae	Chordariaceae		no clear affiliation	LT546282, LT546308	Chordariaceae sp. 2 AP‐2016
BI045	Chordariaceae		Chordariaceae		LT546283	Chordariaceae sp. 6 AP‐2016
BI047	***Pylaiella washingtoniensis***	*Pylaiella washingtoniensis*		*Pylaiella*, not *littoralis*	LT546284, LT546309	
BI048	Chordariaceae	Chordariaceae		Chordariaceae	LT546285, LT546310	Chordariaceae sp. 7 AP‐2016
BI051	***Dictyosiphon*** **sp. 1GWS**	*Dictyosiphon* sp. 1GWS		*Dictyosiphon* sp.	LT546286, LT546311	Morphology: *Dictyosiphon*
BI052	*Stictyosiphon tortilis*		Ectocarpaceae	Ectocarpaceae	LT546287, LT546312	Morphology: *Stictyosiphon tortilis*
BI059	*Hincksia* cluster	*Hincksia* cluster	*Hincksia* cluster	*Hincksia* cluster	LT546288, LT546313	Acinetosporaceae sp. 3 AP‐2016
BI061	***Desmarestia aculeata***	*Desmarestia aculeata*	*Desmarestia aculeata*		LT546289	
BI062	***Agarum clathratum***	*Agarum clathratum*	*Agarum clathratum*		LT546290	
BI063	***Saccharina latissima***	*Saccharina latissima*	*Saccharina latissima*	*Saccharina latissima*	LT546291, LT546314	
BI064	Sister to *Desmarestia viridis*	Desmarestiales	Desmarestiales	Desmarestiales	LT546292, LT546315	*Desmarestia* sp. 1 AP‐2016
BI065	Sister to *Desmarestia viridis*	Desmarestiales		Desmarestiales	LT546293, LT546316	*Desmarestia* sp. 1 AP‐2016
BI067	Chordariaceae			Chordariaceae	LT546317	Chordariaceae sp. 8 AP‐2016
BI068	Chordariaceae			Chordariaceae	LT546318	Chordariaceae sp. 8 AP‐2016
BI077	***Saccharina latissima***	*Saccharina latissima*		*Saccharina latissima*	LT546294, LT546319	
Proportion identified to species	0.41	0.5	0.25	0.32		

**Figure 8:**
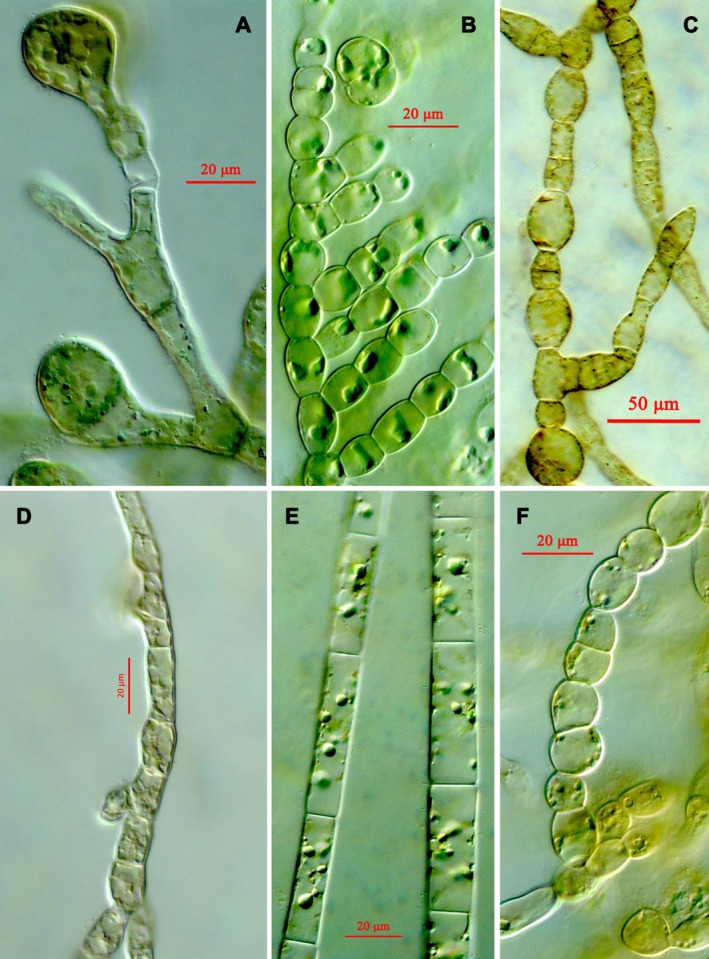
Micrographs of permanent mounts prepared from cultured isolates from substratum samples. (A) Gametophyte of *Saccharina latissima* (BI063); (B) gametophyte of *Desmarestia aculeata* (BI061); (C) gametophyte of a hitherto unsequenced, possibly novel *Desmarestia* sp. (BI064); (D–F) 3 examples of previously unsequenced, filamentous brown algae (D: BI0033, E: BI048, F: BI030).

Sequencing (Figs. 5, 6, 7) revealed two gametophyte isolates of *S. latissima* (Fig. 8A), a gametophyte of *D. aculeata* (Fig. 8B), two gametophytes of a hitherto‐unsequenced Desmarestia (Fig. 8C), a gametophyte of *A. clathratum* , six isolates of two different Pylaiella species (*P. littoralis* and *P. washingtoniensis*), and 10 previously unsequenced, still unidentified brown algae (3 examples: Fig. 8, D–F).

### Seaweed pathogens

The oomycete pathogen *E. dicksonii* was repeatedly observed in the filamentous brown alga *Pylaiella* sp.

## Discussion

The results provide a platform of biological information for the northern Baffin Island region providing a solid baseline for detecting changes in the community composition in the context of ongoing and expectable environmental change. The scope of this study was on macroalgae and the inventory presented here can be considered close to comprehensive, even though a few microalgae (for which there are no surveys at all yet from the Baffin Island region) are reported as well. They constitute a baseline of biodiversity data of important primary producers, and also for future quantitative studies of standing stock composition. The observations reported here cover the sublittoral down to 15 m, which corresponds to the depth zone in which Arctic macroalgae were previously shown to be the major primary producers (Krause‐Jensen et al. 2007). We suggest that the study sites around Cape Hatt/Ragged Channel be used as a reference by future studies for detecting changes in seaweed community composition in the Canadian High Arctic. Previous studies such as the BIOS project (Cross et al. 1987) were incomplete with regard to attached algae and associated eukaryotic pathogens. The 2009 expedition together with the culturing approach using marine sediment/substratum inocula and a substantial backlog of unpublished information available at the University of Massachusetts, Amherst, has provided a more comprehensive synopsis of the marine flora. The 2009 expedition was preceded by changes in water temperature and the complete disappearance of summer sea ice. Sea ice dynamics of the study site at Cape Hatt in the early 1980s are well documented (Dickins 1987), when the area experienced an average 63 days of open water a year with ice break‐up starting in June and freeze‐up starting in late September.

Adey (Adey et al. 2008, Adey and Hayek 2011) used the Thermogeographic Model to explain the strong Pacific affinity of endemic Arctic seaweed diversity, while overall the Arctic seaweed flora had previously been shown to have rather Atlantic affinities (Dunton 1992). The number of known endemic taxa in the Arctic is low and includes *P. glacialis*,* L. solidungula*,* D. socialis*,* Devaleraea ramentacea*,* Turnerella pennyi*,* Pantoneura baerii*, but also the crustose coralline species *Clathromorphum compactum* (Lüning 1990, Adey et al. 2008) – all of which were recorded from the Cape Hatt region within the framework of this study.

### Kelps

Sporophytes of the kelps *S. latissima*,* S. longicruris*,* L. solidungula,* and *A. esculenta* (together with *D. aculeata*) dominated the phytobenthos by forming a dense canopy in high‐current locations between 5 and 10 m depth. Arctic kelps have been shown to support diverse invertebrate communities (Dunton and Schell 1987). Among the invertebrates, only the green sea urchin *Strongylocentrotus droebachiensis* has significance as a grazer of kelp communities in parts of the Arctic – in contrast, the abundant amphipod *Gammarelus hommari* feeds mostly on delicate red algal species like *D. ramentacea* (Wessels et al. 2006). Interestingly, the previous surveys at Cape Hatt had not recorded sea urchins. Kelps attain high standing stock in their communities and they have high rates of light‐saturated photosynthesis and of photosynthesis to respiration at 0°C (Dunton and Dayton 1995). Except for *A. esculenta*, the same kelp species were found, albeit in a much patchier manner and not forming a contiguous canopy, in the calm waters of fjordic locations. The Germling Emergence Method (Peters et al. 2015) confirmed the presence of gametophytes of these kelp species in the substrata in the vicinity of Cape Hatt. This is worth noting since gametophytes of kelps (and of *Desmarestia* species, see below) have rarely been isolated directly from abiotic substratum. Previously, they had been observed as endophytes of red algae (Moe and Silva 1989, Garbary et al. 1999, Hubbard et al. 2004), however, in our Arctic material they developed from sand grains and pebbles.

#### Cryptic macroalgal diversity revealed by the Germling Emergence Method

As in temperate regions (Peters et al. 2015), a bank of microscopic forms was present in the field at the Baffin Island study site. This allowed isolation of a multitude of clones from substratum and subsequent identification by molecular barcoding. Because fewer reference sequences are available for 3′‐COI and 5′‐cox3 than for 5′‐COI, the latter allowed more precise affiliations and identifications (Tables 3, 4).

A majority of taxa isolated belonged to the Ectocarpales, which are annual or ephemeral algae. Also isolated were the gametophytes of the common brown overstory kelps, *A. clathratum* and *S. latissima*, and a gametophyte of the Atlantic genotype 1a (sensu Saunders and McDevit 2013) of *Desmaretia aculeata* (Fig. 8c). Two further isolates (BI064 and BI065) resembled kelp gametophytes (Fig. 5); they consistently grouped with *Desmarestia viridis*, (Fig. 5 to 7) however, the genetic distance of 5% to the latter in 5′‐COI lies beyond the species‐limit cut‐off at 1.3% (Yang et al. 2014) and suggests that they belong to a different, closely related species. Such a putative sister species of *D. viridis* is so far unknown and has not been seen in previous molecular studies; isolates of *D. viridis* from Europe, Japan, and southernmost South America (under the name *D. willii*) differed by just a single nucleotide substitution in ITS (van Oppen et al. 1993). Recollection and a thorough revision of Arctic *D. viridis* specimens appear required to detect the macroscopic sporophyte of this entity. All our kelp and *Desmarestia* gametophytes have remained vegetative and their sex undetermined. None of our isolates belonged to *P. glacialis*, which was common at the sampling site (Kawai et al. 2015b).

Among the variety of strains belonging to the Ectocarpales, there were a few good sequence matches. We isolated four strains of the kelp endophyte *Laminariocolax aecidioides*, originally described from Greenland (Rosenvinge 1893). In 5′‐COI, our strains strongly resembled either a previous isolate from Brittany, or the taxa *L. macrocystis* and *L. eckloniae* described from the southern hemisphere. As discussed in Peters et al. (2015), the genetic distance of *L. macrocystis* and *L. eckloniae* is not sufficient to maintain their taxonomic separation from *L. aecidioides*.

Strain BI051 formed macrothalli in culture and was morphologically identified as *Dictyosiphon*. In 5′‐COI it was 99% identical to the cryptic *Dictyosiphon* sp. 1GWS from Churchill (Saunders and McDevit 2013). Comparison with other taxa of *Dictyosiphon* showed that this species is neither *D. macounii* (Saunders and McDevit 2013) nor *D. chordaria* or *D. ekmanii* (Fig. 5). A further entity detected at Churchill, and referred to as *Dictyosiphon* sp. 3GWS (Saunders and McDevit 2013), appears too far distant genetically to belong to the same genus (Fig. 5).

At Churchill, Saunders and McDevit (2013) discovered a surprising genetic diversity in specimens of *Pylaiella*, including the presence of the Pacific taxon *P. washingtoniensis*. Of our six strains of *Pylaiella*, five also belonged to *P. washingtoniensis*, and one to a European entity of *P. littoralis* (Oudot‐Le Secq et al. 2001, Geoffroy et al. 2015). Strain BI024 showed 2% genetic distance from *P. washingtoniensis* in 5′‐COI. More samples of Arctic *Pylaiella* are required to reveal whether BI024 belongs to yet another species.

The taxa listed so far could be barcoded because of a close similarity to a published sequence. The remaining 17 isolates (11 sequences in each 5′‐COI and 3′‐COI, and 13 in 5′‐cox3), did not match any published sequence, and the identities of these algae remain yet to be uncovered. They showed large genetic distances (8%–12% diversity in 5′‐COI) to the closest published sequence, however, the different markers placed them usually in the same clusters (Table 4). A majority of eleven strains were thus affiliated with Chordariaceae, four with the *Hincksia* cluster, and one in Scytosiphonaceae. None of our isolates grouped with *Acinetospora* (Figs. 5, 6, 7).

Strain BI052 formed macrothalli morphologically identified as *S. tortilis*. We did not succeed in sequencing its 5′‐COI, therefore we used a published sequence of this species (from Churchill, Saunders and McDevit 2013) for our analyses. The three markers consistently placed the species in Ectocarpaceae, well distant from *S. soriferus*, the only other *Stictyosiphon* with public sequences available. The type species, *S. adriaticus*, has apparently not yet been sequenced. The trees suggest phylogenetic affiliation of *S. tortilis* with the Ectocarpaceae, which needs further examination.

For 42 strains obtained with the Germling Emergence Method, no species‐level identification is possible at present even though DNA barcoding reveals their higher level taxonomic affinities or their closest sequenced relatives for which sequences are currently available (Tables 3 and 4). Further investigations will have to reveal whether they are merely unsequenced species, yet previously described from elsewhere, or whether they are genuinely new to science and possibly still undescribed Arctic endemics. Given the strong potential of the Germling Emergence Method to uncover cryptic algal diversity, it will certainly help elucidate composition changes more accurately than macroscopic field collections.

### Pathogens

This study recorded a eukaryotic pathogen affecting seaweeds, *E. dicksonii*, which constitutes a first record for the American Arctic. This suggests that seaweeds in the Arctic may be under similar pressure from eukaryotic pathogens as in temperate latitudes (Küpper and Müller 1999, Gachon et al. 2009). A single record for *Eurychasma* had been made in Svalbard 32 years earlier (Jenneborg 1977).

### Interactions of Arctic seaweeds with the physical environment

The transparency of the water in the Arctic is high for most of the year (up to several tens of metres of visibility), but tends to decrease in the summer and early autumn due to phytoplankton, mixing, and terrestrial runoff (Zacher et al. 2009). Like the terrestrial environment of the high Arctic, the intertidal is characterized by extreme temperature fluctuations, ranging from as low as −55°C in winter to +15°C in the brief Arctic summer. Not surprisingly, hardly any perennial seaweeds survive such extreme environmental conditions and fluctuations. In this context, the observation of *F. evanescens* in what is likely an isolated, ice‐sheltered patch in the intertidal is significant. It is reasonable to hypothesize that with less sea ice, this and other species would colonize higher in the upper subtidal and low intertidal.

For around 8 months a year, the sea in the study area is covered by ice. For 2011 and 2012 the sea ice freeze up was around November 20, when the sea ice became useable with snowmobiles. In 2013 the freeze up happened earlier and became useable around November 12. Breakup usually starts in the first 2 weeks of July, with the sea in 2013 being totally ice free on July 31 (P. Ootoowak, Pond Inlet, pers. comm.). For 2011 and 2012, the sea ice freeze‐up was around November 20, when the sea ice became useable with snowmobiles; in 2013, the freeze‐up happened a bit earlier and sea ice became useable for vehicles around the November 12 (P. Ootoowak, Pond Inlet, pers. comm.). In 1980, freeze‐up in the Pond Inlet/Cape Hatt region usually occurred in early October (Dickins 1987). The absence of any remnants of sea ice during the 2009 expedition is unprecedented and in stark contrast with every previous expedition to the area of Ragged Channel, Cape Hatt, and Eclipse Sound. All previous and including the last expedition to this study site (early September 2003, G. W. Saunders, pers. comm. and R. T. Wilce, unpubl.) encountered broken sea ice for much of the transit from Pond Inlet to Cape Hatt, and inside Z Lagoon and Bay 11.

Changes in sea ice are matched by the temperatures recorded by the temperature logs of the dive teams: temperatures of 0°C–1°C were logged for all dives in 2003 (G. W. Saunders, pers. comm.), contrasting with 2°C in open water and beneath a thermocline in Bay 11 and Z Lagoon in 2009, and up to 7°C near the surface in the sheltered system of Z Lagoon. A surface temperature of 7°C in Z Lagoon seems exceptional. The only available historic records for this site (Buckley et al. 1987) include extensive temperature transects for the same site in early September 1980, which revealed top surface temperatures around 4°C inside Z Lagoon and bottom temperatures inside the Lagoon beneath 1°C, while in Ragged Channel just outside the Lagoon a surface temperature of around 3°C was recorded, falling to 2°C at 10 m and beneath 1°C beyond 30 m depth. Even though patchy, and in line with the accounts of Inuit residents of the area, these observations suggest that the region of northern Baffin Island is not exempt from the loss of sea ice cover observed for the Arctic in general.

### Outlook

Similar to other marine bioregions such as the southwestern Antarctic Peninsula (Mystikou et al. 2014), scientists monitoring the American Arctic phytobenthos are likely confronting the shifting baselines problem (Jackson et al. 2001, Jackson 2008, Knowlton and Jackson 2008), i.e., due to the lack of reliable datasets of community composition prior to the onset of major environmental change it may be difficult to assess the impacts of the change on the community in question. Recent studies (Asensi and Küpper 2012, Tsiamis et al. 2013) have highlighted the value of historic datasets in assessing the changes in seaweed‐dominated coastal ecosystems. In this context, the study by Kortsch et al. (2012) presents an exceptional dataset from Svalbard over a 30‐year period of gradually increasing seawater temperature and decreasing sea ice cover, showing a 5‐ to 8‐fold increase in macroalgal cover in the inshore benthic communities. Indeed, scenarios for a warming Arctic with a shortening period of sea ice cover predict an increase of both benthic and pelagic primary production (e.g., Rysgaard and Glud 2007).

Another interesting observation worth further study is the large amount of dead macroalgal biomass (Fig. 4), which seems to be typical from the sheltered water bodies of High Arctic fjords. Despite the increased interest in blue carbon sequestration worldwide – in particular, in seagrass meadows, salt marshes, and mangroves (Duarte et al. 2013, Hyndes et al. 2014), nothing is presently known about the role of coastal marine macrophyte communities from Polar Regions. The extent, functioning, and carbon residence time of these fjordic macrophyte communities should be explored in this respect.

This project was supported by SAMS and NFSD core funding (Oceans 2025 WP 4.5 from the UK Natural Environment Research Council), the European Commission (ASSEMBLE, grant agreement no. 227799), and the TOTAL Foundation (Paris; Project “Macroalgal and oomycete benthic diversity in the Canadian Marine Arctic”). This work also received funding from the MASTS pooling initiative (The Marine Alliance for Science and Technology for Scotland) and their support is gratefully acknowledged. MASTS is funded by the Scottish Funding Council (grant reference HR09011) and contributing institutions. We also would like to thank Laura Grenville‐Briggs (KTH, Stockholm) for help with bioinformatics analyses as well as Cindy Grant and Philippe Archambault (University of Quebec, Rimouski) for help with preparing the map of the study area (Fig. 1).
